# The Emerging Role of Circulating Tumor DNA in the Management of Breast Cancer

**DOI:** 10.3390/cancers13153813

**Published:** 2021-07-29

**Authors:** Mira Shoukry, Sacha Broccard, Jamie Kaplan, Emmanuel Gabriel

**Affiliations:** Department of Surgery, Section of Surgical Oncology, Mayo Clinic, Jacksonville, FL 32224, USA; Broccard.Sacha@mayo.edu (S.B.); Kaplan.Jamie@mayo.edu (J.K.); Gabriel.Emmanuel@mayo.edu (E.G.)

**Keywords:** circulating tumor DNA, liquid biopsy, breast cancer, recurrence, chemotherapy

## Abstract

**Simple Summary:**

As breast cancer diagnoses continue to rise among women worldwide, it is important to explore and improve upon methods of diagnosis and surveillance. Liquid biopsies have the potential to become the forefront of such efforts, with circulating tumor DNA (ctDNA) increasingly being studied as a biomarker for breast cancer. This review aims to summarize the current applications of ctDNA in the diagnosis and surveillance of breast cancer. Additionally, comparisons between ctDNA and other currently utilized methods and biomarkers provide further insight into the emerging role of ctDNA for the management of breast cancer.

**Abstract:**

With the incidence of breast cancer steadily rising, it is important to explore novel technologies that can allow for earlier detection of disease as well more a personalized and effective treatment approach. The concept of “liquid biopsies” and the data they provide have been increasingly studied in the recent decades. More specifically, circulating tumor DNA (ctDNA) has emerged as a potential biomarker for various cancers, including breast cancer. While methods such as mammography and tissue biopsies are the current standards for the detection and surveillance of breast cancer, ctDNA analysis has shown some promise. This review discusses the versatility of ctDNA by exploring its multiple emerging uses for the management of breast cancer. Its efficacy is also compared to current biomarkers and technologies.

## 1. Introduction

Breast cancer remains the most commonly diagnosed cancer and the leading cause of cancer deaths among women worldwide [1]. In the United States alone, the American Cancer Association predicts that approximately 281,550 new cases of breast cancer will be diagnosed in 2021, with an estimated 43,600 deaths [2]. While the incidence of female breast cancer continues to increase, the mortality rate has decreased by 1% annually since 2013 [2]. Although these trends are primarily thought to be the result of increased screening and awareness, they can also be credited to several recent advances in the approach to breast cancer diagnosis, treatment, and surveillance. Early diagnosis appears to be key to achieving improved treatment decisions and patient outcomes. Thus, research studies have emphasized the importance of accurate biomarkers for the early detection of breast cancer.

One promising area of research is the use of circulating tumor DNA (ctDNA) in the diagnosis and surveillance of breast cancer. While the concept of cell-free DNA has been around for many decades, ctDNA remains a heavily researched topic since its discovery in the 1970s. Although all individuals have cell-free DNA, cancer patients have been found to have higher amounts of cell-free DNA, some of which was deemed to be tumor-derived ctDNA [3]. The mechanisms by which the tumor DNA reaches the bloodstream are still being debated, and the precise differentiation between circulating non-tumor DNA and tumor-specific ctDNA remains a significant challenge. Among several proposed mechanisms, the main source of ctDNA is thought to be from the phagocytosis of necrotic and apoptotic tumor cells by macrophages and the subsequent release of their digested DNA [4]. The ctDNA fragments vary greatly in base pair sizes, and their rate of release is likely correlated with their respective tumor characteristics from which they derive, including size, tumor burden, and location [5]. However few and small, these fragments carry genetic mutations identical to the tumor itself and may even be capable of triggering oncogenic changes in healthy surrounding cells through a process termed genometastasis, which refers to the transfer of mutated DNA from cancer cells into normal (non-tumor) cells that are remote from the tumor [6,7,8].

A myriad of factors makes ctDNA an appealing biomarker in the diagnosis and surveillance of several cancers. Unlike other tissue biomarkers, ctDNA is identified through “liquid biopsies” as opposed to standard tumor tissue biopsies. Whereas tissue biopsies may be invasive and sometimes difficult to reproduce, liquid biopsies may detect ctDNA through non-invasive means by simply obtaining blood [6]. Existing literature has detailed the use of ctDNA as an indicator of tumor burden. The level of ctDNA in the plasma of cancer patients has been found to correspond to different stages of cancer such that late-stage patients displayed higher levels of ctDNA in their plasma when compared to early-stage patients [9]. In addition to its ease of detection, ctDNA fragments are thought to have a short half-life, which may allow for monitoring of tumor progression in a real-time clinical setting [4].

Due to the heterogeneity of tumors, tissue biopsies generally provide information specific to the area from which the biopsy was taken rather than the tumor as a whole. On the other hand, the analysis of ctDNA through liquid biopsies may provide a more comprehensive view of the tumor characteristics, including molecular variations [10]. Due to the feasibility and repeatability of liquid biopsies, ctDNA may be used to identify mutations early on. Ongoing trials may determine the correlation between mutations within the primary tumor and those found within ctDNA, such as the French-led pilot study NCT04104633 (Feasibility Study of a Molecular Karyotype Using a Very High-throughput Sequencing Approach, the “Massive Parallel Sequencing” on Circulating Tumor DNA). This early study plans to enroll 10 patients with breast cancer and perform molecular karyotyping from ctNDA for comparison with whole genome sequencing on the primary tumors from corresponding patients.

While ctDNA is a relatively novel concept, there seem to be emerging uses of ctDNA in the management of breast cancer. This review discusses the progressing role of ctDNA in the diagnosis and surveillance of breast cancer and how it compares to existing methods and technology.

## 2. The Role of ctDNA in Breast Cancer

### 2.1. ctDNA in the Diagnosis and Early Stages of Breast Cancer

Mammography partnered with breast tissue biopsies remain the current standards of care for the diagnosis of breast cancer. However, imaging modalities have variable sensitivity and accuracy in the detection of early breast cancer. Additionally, tissue biopsies are invasive and sometimes difficult to schedule or repeat. As an alternative, ctDNA obtained through liquid biopsies has emerged as a potential biomarker capable of detecting breast cancer, even in early stages (Table 1). Levels of plasma DNA alone may differentiate healthy patients from those with breast cancer. The earliest report of such use was in 1977, in which radioimmunoassay was used to measure ctDNA levels in healthy patients and compare them to those with various cancers including breast cancer [3]. The authors found that ctDNA levels greater than 50 ng/mL were more common among breast cancer patients when compared to control patients. However, the sensitivity of radioimmunoassay-detected ctDNA in identifying breast cancer was only about 38% at the time, and thus, it was deemed a weak diagnostic tool [3]. Huang et al. later demonstrated that breast cancer patients had a significantly higher concentration of plasma DNA (65 ng/mL) when compared to the healthy control patients (13 ng/mL), with 93.4% sensitivity as detected by real-time polymerase chain reaction (PCR) [11].

The size of ctDNA has also been examined as a method of detecting breast cancer. However, there is some debate regarding the consistency of this method. While some groups have shown that larger fragments are more common in cancer patients, others have concluded that ctDNA is in fact more fragmented in cancer patients [12,13]. More recent research efforts have focused on a more personalized approach to ctDNA and its role in breast cancer diagnosis and prognosis. PCR-based methods such as BEAMing (Beads, Emulsion, Amplification, and Magnetics) have gained popularity due to their ability to isolate and amplify tumor-specific mutations with a high degree of sensitivity. As described by Vogelstein and colleagues, BEAMing comprises the process of quantifying DNA sequences by linking them to fluorescently labeled magnetic particles [14]. As tumor protein p53 (TP53) and phosphatidylinositol-4,5-bisphosphate 3-kinase catalytic subunit alpha (PIK3CA) are among the most commonly mutated genes in primary and metastatic breast cancer, ctDNA has shown effectiveness in detecting such mutations early on in the disease process [15]. For instance, Madic et al. successfully utilized next-generation sequencing in order to detect TP53 mutations in 81% of patients with a confirmed diagnosis of triple-negative breast cancer [16]. However, when compared to circulating tumor cells, ctDNA mutation frequency showed no prognostic impact on overall survival. Similarly, Beaver et al. reported a sensitivity of 93.3% and specificity of 100% among a small cohort (29 patients) with PIK3CA mutations [17]. Other groups have investigated additional mutations among ctDNA as biomarker targets for the detection of early breast cancer, including 5-Hydroxymethylcytosine, MDM2 and MDM4, and AKT1 [18,19,20].

When compared to standard tumor tissue biopsies, ctDNA may reveal critical information to provide an earlier and more accurate diagnosis of breast cancer. Higgins et al. used both sequencing and BEAMing to screen for PIK3CA mutations in plasma samples of breast cancer patients. Not only did BEAMing identify breast cancer with 100% accuracy, but the ctDNA results were also comparable to those obtained by sequencing archival tumor tissue DNA [21]. The study concluded that mutational analysis using ctDNA may be a stronger biomarker than archival biospecimens, as it is more sensitive to PIK3CA mutational status changes. A similar study by Rodriguez et al. used next-generation sequencing on matched plasma samples and fresh tissue biopsies from patients with primary breast cancer and found additional TP53 and PIK3CA mutations in ctDNA, which were not identified in the tissue biopsies [22]. Furthermore, a study by Board et al. explored the sensitivity of PIK3CA mutations in ctDNA to assess localized versus metastatic breast cancer. While PIK3CA mutations were identified in the tumor tissue of 47% of patients with localized breast cancer, no mutations were detected in the matched ctDNA. Conversely, PIK3CA mutations were successfully detected in the plasma-derived ctDNA of patients with metastatic disease with a 95% concordance when analyzed parallel to matching tumor tissue DNA [23]. These studies indicate that plasma ctDNA may be used as an accurate alternative to tumor tissue data, especially when the latter cannot be readily collected.

Ongoing prospective studies will continue to evaluate whether ctDNA can be used in real time to diagnosis breast cancer in early stages. A number of new technologies are being developed to determine whether ctDNA analysis can lead to early diagnosis. For example, the French trial ICRG0101 (Interest of Broadband Spectroscopy Analysis by Infrared Laser on Liquid Biopsies in Breast Cancer Screening) aims to determine the feasibility of broadband laser spectroscopy on liquid biopsies for breast cancer screening (NCT04273542). This is a large, multi-center trial planning to enroll 1100 participants, and therefore, it should be powered well to determine the prognostic utility of ctDNA for the diagnosis of early-stage breast cancer.

**Table 1:** Summary of studies exploring the use of ctDNA for the diagnosis of breast cancer.

Reference	Study Design	Method of ctDNA Analysis	Results
Leon et al. [3], 1977	228 patients	Radioimmunoassay	- Cancer patients showed higher concentrations of serum ctDNA when compared to healthy controls - A decrease in serum ctDNA levels was observed in patients responding to radiation therapy while unsuccessful treatment yielded unchanged or increased ctDNA levels
	(173 with breast cancer, 55 control)		
Huang et al. [11], 2006	121 patients	PCR	- Median concentration of plasma ctDNA was 13 ng/mL in healthy controls compared to 65 ng/mL in patients with malignant breast cancer - ctDNA levels were significantly reduced following surgical resection
	(94 with breast cancer, 27 control)		
Agostini et al. [12], 2012	88 patients	PCR	- Greater amounts of large ctDNA fragments (ALU247) were found in breast cancer patients with regional lymph node metastasis compared to small fragments (ALU115)
	(39 with breast cancer, 49 control)		
Madhavan et al. [13], 2014	383 patients	PCR	- Reduced ctDNA integrity was correlated with primary and metastatic breast cancer - Increased progression-free and overall survival were correlated with increased ctDNA integrity
	(283 with breast cancer, 100 control)		
Madic et al. [16], 2015	40 patients	Next-generation sequencing	- TP53 were identified in the ctDNA of 81% of triple-negative breast cancer patients - ctDNA showed weaker prognostic value when compared to circulating tumor cells
	(All with triple-negative breast cancer)		
Higgins et al. [21], 2012	49 patients	BEAMing	- A concordance of 100% was found for PIK3CA mutation status by BEAMing of ctDNA and sequencing of tumor tissue
	(All with breast cancer)		
Rodriguez et al. [22], 2019	29 patients	Next-generation sequencing	- Matching TP53 and PIK3CA mutations were observed in both plasma ctDNA and tumor tissue - 4 additional TP53 and PIK3CA mutations, identified using ctDNA, were not previously identified in tumor tissue biopsies
	(All with breast cancer)		
Board et al. [23], 2010	76 patients	Amplification Refractory Mutation System allele-specific PCR + Scorpion probes	- 95% concordance for PIK3CA mutations was found between matched plasma ctDNA and tissue biopsies - PIK3CA mutations were identified in the ctDNA of patients with metastatic disease but not in those with localized disease
	(46 with metastatic breast cancer, 30 with localized breast cancer)		

### 2.2. ctDNA to Assess Response and Resistance to Treatment

Analyzing ctDNA levels may also provide a biomarker by which treatment response or resistance may be assessed. Currently, serial serum levels of cancer antigen 15-3 (CA 15-3) are considered adequate representations of breast cancer treatment efficacy [24,25,26]. However, some investigators caution the use of CA 15-3 levels as the sole measure of therapy response due to its low sensitivity (60–70%) and sporadic spikes during the course of treatment [27,28]. Comparatively, changes in ctDNA have been shown to correspond with response to treatment in breast cancer patients (Table 2). In fact, when compared to cancer antigen 15-3, a prospective study by Dawson et al. demonstrated that ctDNA serum levels (sequenced for PIK3CA and TP53 mutations) were significantly more sensitive to changes in tumor burden [29]. Serial changes in ctDNA also corresponded to treatment responses detected on computed tomography (CT) imaging. Furthermore, an increase in ctDNA levels indicated progressive disease an average of 5 months prior to its discovery on imaging [29].

Existing literature has highlighted the use of ctDNA as a biomarker for response to various chemotherapeutic drugs, such as palbociclib, fulvestrant, and bevacizumab, among others [30,31]. For instance, a study by Chen et al. characterized the correlation between ctDNA levels and treatment efficacy in breast cancer patients. It was shown that HER2+ breast cancer patients who had developed resistance to trastuzumab showed significantly higher levels of HER2/ERBB2 in their ctDNA when compared to those who benefitted from the treatment [32]. Additionally, HER2- breast cancer patients with resistance to chemotherapy showed genetic variations in the TP53, PIK3CA, and DNA damage repair genes. A more recent study by Magbanua et al. found that the detection of ctDNA corresponded to a worse probability of achieving complete pathological response (cPR) to neoadjuvant chemotherapy in breast cancer patients. Of note, the study also found a correlation between ctDNA clearance and improved survival in patients who did not necessarily achieve cPR to treatment [33]. In a study by Riva et al., serial levels of ctDNA containing TP53 mutations corresponded with response to neoadjuvant chemotherapy with 100% accuracy in patients with nonmetastatic, triple-negative breast cancer [34]. Additionally, in metastatic breast cancer patients undergoing multimodal treatment, ctDNA may be utilized to predict drug resistance and disease progression. This was demonstrated in a study by Hu et al. where a significantly increased frequency of mutations to the PIK3CA, TP53, NOTCH2, MLL3, and SETD2 genes was seen in triple-negative breast cancer patients exhibiting drug resistance following 3 months of chemotherapy [35]. ctDNA was also found to reflect disease progression due to such resistance earlier than standard imaging [35].

ctDNA has also been found to be sensitive to epigenetic changes. A study by Sharma et al. explored the use of tumor-specific ctDNA to detect methylation changes in response to neoadjuvant chemotherapy in breast cancer patients. Of the genes analyzed, the methylation frequency of BRCA1 was found to be significantly reduced only in those responding to neoadjuvant treatment [36]. Additional methylation trends were observed in a study by Takahashi et al., in which significantly decreased levels of methylated ctDNA corresponding to the RASSF1A promoter gene were found in breast cancer patients responding to neoadjuvant chemotherapy compared to those that did not. Furthermore, methylated ctDNA was found to be a more sensitive indicator of treatment response when compared to carcinoembryonic antigen as well as cancer CA 15-3 [37]. Similarly, Avraham et al. found undetectable RASSF1 methylation to be associated with complete pathological response to neoadjuvant chemotherapy [38].

Ongoing prospective studies will evaluate whether ctDNA can be used in real time to predict response to therapy. The Liquid Biopsies and Imaging in Breast Cancer (LIMA) trial led by UMC (Universitair Medisch Centrum) Utrecht in the Netherlands will combine multi-parametric MRI imaging obtained during neoadjuvant chemotherapy with ctDNA from 100 patients with breast cancer (NCT04223492). The study’s objective is to develop a predictive model, which combines imaging data and genetic data derived from ctDNA, that estimates response to neoadjuvant chemotherapy. Another study led by Centre Oscar Lambret, France, aims to determine whether ctDNA can be used to track response to the CDK4/6 inhibitor palbociclib in patients with hormone receptor positive (HR+), HER2- metastatic breast cancer. (NCT04653740). This single-arm pilot study, OMERIC (Omic Technologies to Track Resistance to Palbociclib in Metastatic Breast Cancer), aims to characterize molecular changes associated with resistance to palbociclib at the individual patient level.

### 2.3. ctDNA for Early Detection of Recurrence

Despite best efforts to minimize residual disease following treatment, recurrence of breast cancer is common. While physical examination and imaging (mammogram, MRI) are the current standards in post-treatment surveillance of breast cancer, some recurrences remain undetected [39,40]. This inaccuracy becomes more apparent with computer-aided detection (augmented with artificial intelligence), which has been shown to improve the overall accuracy and diagnostic specificity of mammography compared to non-computer-aided detection [41]. In addition, applications of artificial intelligence have been applied to ultrasound (US) imaging in order to better detect breast cancer [42,43]. Novel techniques in high-resolution, low frequency US and neural networks to analyze densely pixelated images have been reported to correlate with radiologist diagnostic interpretations of surveillance imaging for patients with breast cancer.

Artificial intelligence is also being applied to the analysis of liquid biopsies for cancer, including those with breast cancer [44]. An active clinical trial, TRICIA (TRIple Negative Breast Cancer Markers In Liquid Biopsies Using Artificial Intelligence) led by the Jewish General Hospital in Montreal, Quebec, Canada, seeks to test novel informatics tools to develop a test or score for patients with triple negative breast cancer based on the expression of ctDNA within the patient cohort (NCT04874064). This test will be used to predict response and recurrence to neoadjuvant chemotherapy. The group plans to enroll 130 patients and perform whole exome sequencing data from the ctDNA, use machine-learning algorithms to integrate the ctDNA data with clinical outcomes, and develop a biomarker score that may identify those patients with triple negative breast cancer who may not respond to neoadjuvant chemotherapy or have a recurrence. In this way, selected patients may be able to be spared from the toxicities of chemotherapy, specifically if their tumors are not predicted to achieve a response based on their ctDNA analysis.

As ctDNA may represent tumor-specific mutations as they occur, it may be used as an alternative or as a complement to standard imaging surveillance in order to improve early detection of recurrence. For example, a retrospective study by Olsson et al. analyzed ctDNA derived from the plasma of breast cancer patients (Table 3). The authors showed that 93% of patients who developed metastasis showed evidence of tumor-specific ctDNA up to 3 years prior to clinical detection. Conversely, ctDNA was not detected in patients with long-term disease-free survival [45]. Similarly, a more recent study by Coombes et al. explored the efficacy of personalized ctDNA profiling in the detection of breast cancer recurrence compared to standard clinical and radiological surveillance. Notably, ctDNA tests were able to detect recurrence an average of 8.9 months ahead of clinical surveillance in 89% of relapsed patients [46]. Such early identification of recurrence may allow for more effective and possibly life-saving treatments before the tumor recurrence progresses further.

The presence of ctDNA in the plasma, or the lack thereof, may also be used to predict disease-free survival. In a study reported by Radovich et al., the presence of ctDNA following neoadjuvant chemotherapy was significantly correlated with a poorer distant disease-free survival and overall disease-free survival among patients with triple-negative breast cancer [47]. Thus, similar to its use in the diagnosis of breast cancer, mutation tracking with ctDNA may also be utilized to predict recurrence. Through digital polymerase chain reaction (dPCR), Garcia-Murillas et al. reported a correlation between the post-surgical increase in PIK3CA mutated ctDNA with increased incidence of recurrence. This study also found that the detection of ctDNA following treatment predated clinical identification of relapse by a median of 7.9 months [48]. The aforementioned studies highlight how the analysis of ctDNA through plasma samples may precede clinical methods of detecting breast cancer recurrence, thus potentially allowing for more efficient treatment decision making.

## 3. Conclusions

There remains an unmet need for new technologies that allow physicians to better treat patients with breast cancer. Liquid biopsies and ctDNA in particular are potentially bringing health care providers closer to that goal. Novel techniques such as BEAMing and next-generation sequencing have also made it easier to analyze breast cancer on a more personalized genomic level. However, further research into the clinical use of ctDNA is warranted, as the majority of existing studies are retrospective in nature. While they may lack homogeneity in their methods, these studies have demonstrated some benefits of using ctDNA for the early diagnosis of breast cancer, the monitoring of disease progression and response to treatment, and as a potential predictor or recurrence. In addition to the tremendous advancement over recent years, the emerging role of ctDNA in the management of breast cancer will continue to be explored and has the potential to expand even further through ongoing prospective studies.

## Figures and Tables

**Table 2:** Summary of studies exploring the use of ctDNA for the assessment of treatment response in breast cancer patients.

Reference	Study Design	Method of ctDNA Analysis	Results
Dawson et al. [29], 2013	30 patients	Tagged-amplicon deep sequencing or paired-end whole-genome sequencing	- Levels of PIK3CA and TP53 mutations in ctDNA reflected changes in tumor burden in response to treatment long before they were seen on imaging - ctDNA more accurately reflected tumor progression when compared to CA 15-3
	(All receiving systemic treatment)		
Darrigues et al. [30], 2021	25 patients	Droplet digital PCR	- ctDNA clearance following treatment with palbociclib and fulvestrant was associated with longer progress-free survival - All patients with increased ctDNA levels following treatment showed disease progression
	(All with ER+ HER2− metastatic breast cancer)		
Nakagomi et al. [31], 2017	Case report,	Next-generation sequencing	- Changes in TP53 mutation status as observed through ctDNA were indicative of real-time responses to bevacizumab and paclitaxel
	1 patient		
Chen et al. [32], 2020	31 patients	Targeted next-generation sequencing	- HER2+ breast cancer patients showed increased ERBB2 copy numbers when resistant to trastuzumab - Genetic alterations to the TP53, PIK3CA, and DNA damage repair genes were observed in HER2- patients with resistance to treatment
	(19 HER2+ patients, 12 HER2− patients)		
Magbanua et al. [33], 2021	84 patients	Ultra-deep sequencing	- 100% of patients who achieved pathologic complete response were negative for ctDNA following neoadjuvant chemotherapy - ctDNA-negative patients showed improved survival outcomes regardless of whether or not pathologic complete response was achieved
	(All with breast cancer)		
Riva et al. [34], 2017	46 patients	Droplet digital PCR	- A rise in ctDNA levels during neoadjuvant chemotherapy was correlated with tumor progression - Patients with detectable ctDNA following treatment were found to have poorer disease-free as well as overall survival
	(All with non-metastatic triple-negative breast cancer)		
Hu et al. [35], 2018	68 patients	Next-generation sequencing	- Progression within 3 months of treatment in HR+ patients was correlated with increased TERT, FAT1, and NOTCH4 mutations - Progression between 3–6 months of treatment in HR+ patients was correlated with increased PIK3CA, TP53, MLL3, ERBB2, NOTCH2, and ERS1 mutations
	(All receiving multiline treatment)		
Sharma et al. [36], 2012	30 patients	Next-generation sequencing	- The frequency of BRCA1 methylation was significantly reduced in patients responding to neoadjuvant chemotherapy
	(All with breast cancer)		
Takahashi et al. [37], 2017	87 patients	One-step methylation-specific PCR	- A significant reduction in methylated RASSF1 ctDNA was observed in patients who responded to neoadjuvant chemotherapy but not in those who did not - Methylated ctDNA was found to be a more sensitive biomarker for treatment response when compared to carcinoembryonic antigen and CA 15-3
	(All with primary breast cancer stage II–III)		
Avraham et al. [38], 2012	52 patients	Methylation-sensitive PCR + high-resolution melting	- Undetectable RASSF1 methylation was correlated with complete pathologic response - RASSF1 methylation persisted in patients with minimal or no response during chemotherapy
	(All receiving neoadjuvant chemotherapy)		

**Table 3:** Summary of studies exploring the use of ctDNA for predicting breast cancer recurrence.

Reference	Study Design	Method of ctDNA Analysis	Results
Olsson et al. [45], 2015	20 patients	Droplet digital PCR	- ctDNA accurately identified 93% of patients with eventual clinically detected recurrence and 100% of patients without recurrence - ctDNA evidence of recurrence was identified an average of 11 months prior to clinical detection of metastasis
	(All with primary breast cancer post resection)		
Coombes et al. [46], 2019	20 patients	Ultra-deep sequencing	- Evidence of relapse was detected in plasma ctDNA up to 2 years prior to clinical or radiologic discovery
	(All with primary breast cancer post resection + adjuvant therapy)		
Radovich et al. [47], 2020	196 patients	Next-generation sequencing	- The detection of ctDNA following neoadjuvant chemotherapy was associated with reduced distant disease-free survival and disease-free survival in patients with triple-negative metastatic breast cancer
	(All with triple-negative breast cancer post neoadjuvant therapy)		
Garcia-Murillas et al. [48], 2015	55 patients	Digital PCR	- An increase in ctDNA following surgery was correlated with increased probability of breast cancer recurrence - ctDNA detected recurrence an average of 7.9 months prior to clinical discovery
	(All receiving chemotherapy)		
